# Early diagnosis of airway closure from pigtail signature capnogram and its management in intubated small infants undergoing general anaesthesia for surgery

**DOI:** 10.4103/0019-5049.68379

**Published:** 2010

**Authors:** Sanghamitra Mishra

**Affiliations:** Department of Anaesthesiology and Critical Care, I.M.S & SUM Hospital, BBSR, Orissa, India

**Keywords:** Airway closure, continuous positive airway pressure end tidal carbon dioxide, glottic closure, positive end expiratory pressure, pigtail capnogram, hypercarbia, hypoxaemia

## Abstract

Spontaneous glottis closure during expiration in infants is a normal protective reflex that helps prevent alveolar and small airway collapse (due to compliant chest wall) and thereby maintains functional residual capacity. Endotracheal intubation eliminates this protective mechanism and puts the infant into the risk of hypoxaemia and hypercarbia. This report sums up the early detection of airway closure in a series of three intubated small infants undergoing surgery with general anaesthesia, by the appearance of typical pigtail shaped capnogram, associated with decreased end tidal carbon dioxide and mild hypoxaemia, which was successfully managed by early institution of positive end expiratory pressure.

## INTRODUCTION

In neonates and small infants, due to very compliant chest wall and small diameter airways, there is a tendency for alveolar collapse and small airways closure during expiration, the ultimate implications being reduced functional residual capacity (FRC), ventilation perfusion (VQ) mismatch and decreased CO_2_ excretion/decreased end tidal carbon dioxide (ETCO _2_), leading to hypercapnia and hypoxaemia and acidosis. But this collapse during expiration is usually prevented by “spontaneous *glottic* closure reflex”, a protective reflex normally present in neonates and small infants, thereby maintaining FRC, which improves alveolocapillary gas exchange by increasing the lung volume. Endotracheal intubation (i.e. bypassing the upper airways) eliminates this protective mechanism and puts the infant into the risk of airway closure and alveolar collapse leading to hypercarbia and hypoxaemia.[1–4] Intra operative airway collapse in an infant needs to be diagnosed as early as possible to avoid serious consequences of hypercapnoea and hypoxaemia. This report sums up the incidental early detection of airway closure from *typical pigtail shaped signature capnogram*and therefore timely management by early application of positive end expiratory pressure (PEEP) in a series of three endotracheally intubated small infants undergoing surgery under general anaesthesia.

## CASE REPORT

A 2-month-old female baby weighing 6 kg was posted for congenital umbilical hernia repair under general anaesthesia. The baby had a history of premature birth by 1 month. All investigations were within normal limits. There was no history of recent upper respiratory tract infection.

After usual fasting guidelines, the baby was taken to the operation theatre and intravenous line was secured under light Halothane anaesthesia, after all standard monitors (pulse oximetry, noninvasive blood pressure, precordial stethoscope and ECG) were connected and baseline parameters were recorded, (pulse 112/min, BP 106/66 mmHg and SpO_2_ 100%).

The baby was premedicated with i.v. Atropine 0.01 mg/kg, Ondansetron 0.1 mg/kg and Fentanyl 1.5 mg/kg. Induction was carried out with Propofol 2 mg/kg and intubation was done with Veccuronium 1.2 mg/kg using 3.5 size uncuffed endotracheal tube.

Anaesthesia was maintained with 0.6–1% Isoflurane, 50% N _2_ O, intermittent Vecuronium and Fentanyl. Intermittent positive pressure ventilation (IPPV) was done manually with Jackson-Rees’ circuit in Datex-Ohmeda Aespire s/5 anaesthesia machine with a respiration rate of 20/min and an approximate tidal volume of 10 ml/kg with visible chest-rise and breath sounds audible over all the zones of lung field. After confirming the tube position by auscultation, side stream capnometry was attached and baseline ETCO_2_ of 28 mmHg was recorded.

But after a few minutes, a *typical pigtail shaped signature capnogram* appeared. ETCO_2_ also started falling and reached 18 mmHg. It was also associated with tachycardia (HR 170/min) and fall in SpO_2_ to 89%. The following conditions were excluded one by one.

1. Bronchospasm: as there was no ronchi;
2. VQ mismatch due to atelectasis: ventilated with higher tidal volume, but the condition persisted;
3. Differential lung ventilation: excluded by tube repositioning and equal intensity of breath sound in both sides; and
4. Air leak: excluded by inspection of sampling line/filter, loose connection, etc.

Ultimately, peripheral alveolar collapse and small airway closure (due to elimination of protective glottis closure reflex as a result of endotracheal intubation) was thought to be the cause of typical appearance of pigtail capnogram and associated decrease in ETCO_2_and SpO_2_.

**Figure 1:**
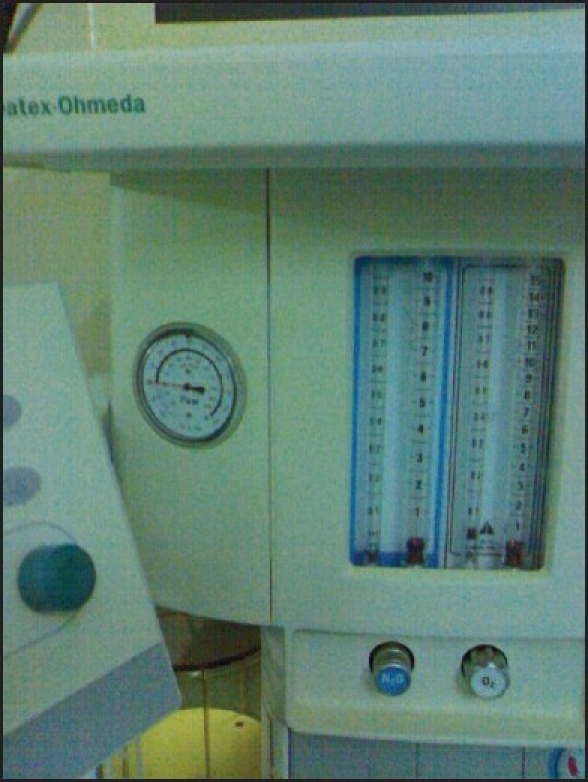
Datex-Ohmeda Aespire s/5 anaesthesia machine showing airway pressure gauze to monitor different airway pressures

Immediately, PEEP was applied manually, keeping maximum airway pressure within 30–35 cm of H_2_O in the airway pressure gauze of the anaesthesia machine[Figure 1].

With the application of PEEP, gradually the pigtail capnogram reverted back to normal and other parameters (ETCO_2_, HR, and SpO_2_) also normalized to baseline values. Again, when PEEP was withheld, the same problem reappeared. Hence, PEEP was continued throughout the ventilation.

Extubation and recovery was uneventful. After recovery, the baby was maintaining oxygen saturation at 98% without oxygen and at 100% with oxygen mask.

After this case report, though it was not an institutional practice to use capnometry, in all paediatric cases, we started using it in all small infants undergoing intubation for surgery.

Over the next 8-month period, two similar cases were recorded. These two were infants undergoing surgery for umbilical hernia (3 months) and meningocele (2 months) and had a history of premature birth by 1 month.

## DISCUSSION

Differential diagnoses of pigtail shaped capnogram involve the following.[5]

**Figure 2:**
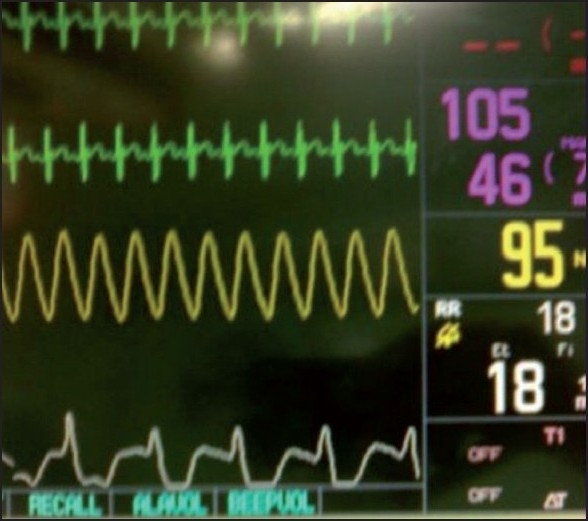
Pigtail signature capnogram (In later part of expiration, excessive recoil of compliant chest wall compresses the alveoli, resulting in a pigtail appearance in third phase of capnogram. This is followed by premature airway closure with abrupt termination of CO2 excretion.)

**Figure 3:**
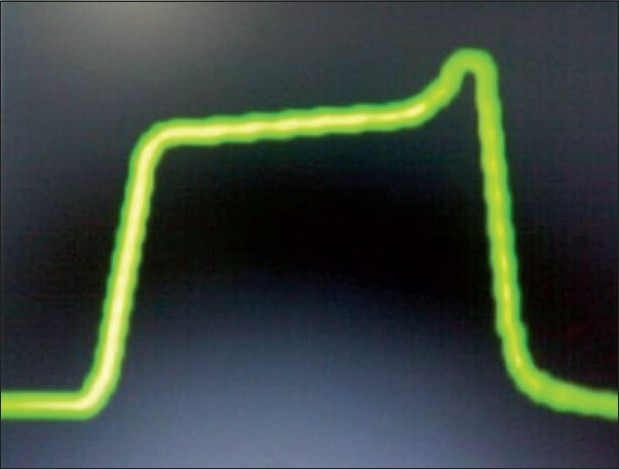
Slit sampling tubes can result in a pigtail capnogram

**Figure 4:**
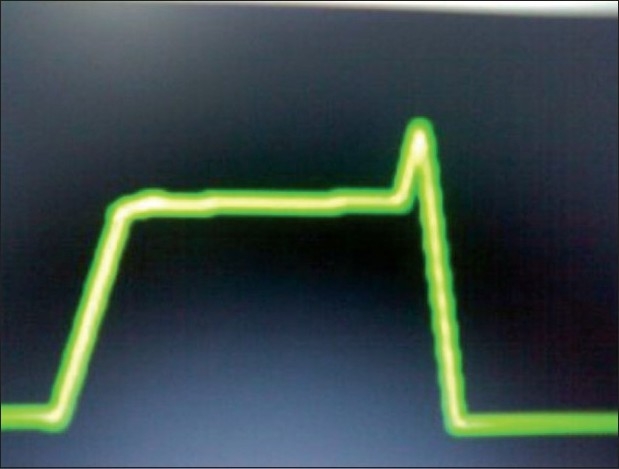
A terminal upswings at the end of phase 3, known as phase 4, can occur in pregnant, obese subjects and low compliance state

**Figure 5:**
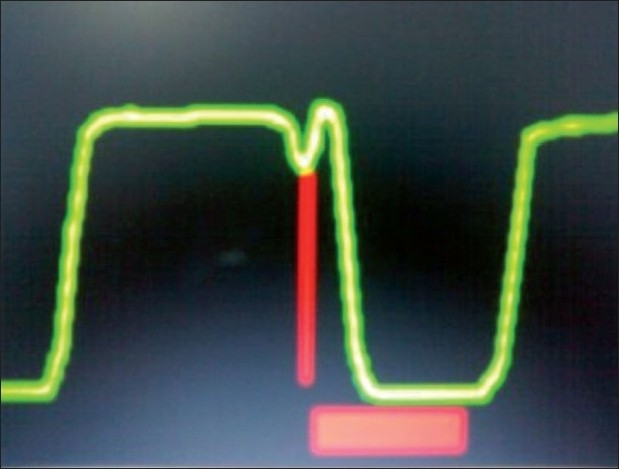
Sticking inspiratory valve – inspiratory flip – horizontal bar indicates possible rebreathing

1. End inspiratory airway collapse in small infants (as in our case)[Figure 2]
2. Air leak in sampling line [Figure 3]
3. Pregnancy/obese [Figure 4]
4. Sticking inspiratory valve [Figure 5]

In neonates and small infants, endotracheal intubation abolishes spontaneous glottis closure reflex required to generate intrinsic PEEP to prevent alveolar collapse and premature small airway closure, due to recoil of their very compliant chest wall.[1–4]

If this continues, alveolar recruitment may not be complete with the usual mode of IPPV and results in decreased FRC, increased VQ mismatch and decreased CO_2_ excretion, leading to hypoxaemia, hypercarbia and associated tachycardia. Infants, especially small ones, tolerate these very poorly.[6]

Upon early detection from *pigtail signature capnogram* and other mentioned parameters, this problem was addressed in time, by application of PEEP, keeping maximum airway pressure within 30–35 cm H_2_O, as there is high cardiovascular tolerance by the infants to increased airway pressure (in comparison to adults) and most infants need peak inspiratory pressure of 25–35 cm H_2_O to achieve adequate ventilation.[7] The mechanism of pigtail appearance in the third phase of capnogram is due to transient increase in expulsion of alveolar air due to sudden alveolar compression by the highly compliant chest wall of the infant. This is followed by compression of terminal airways resulting in premature and abrupt termination of expiration (fourth phase of capnogram) giving rise to low ETCO_2_ value[Figure 2].

History of premature birth might have a bearing on this[8] like postoperative apnoea after general anaesthesia.

## Limitation of the study

If an arterial blood gas (ABG) could have been done during the episode, it would have given a clearer picture on the degree of hypoxaemia and hypercarbia. To conclude, endotracheal intubation, by abolishing spontaneous glottis closure reflex, puts the Infant into the risk of airway closure and alveolar collapse leading to hypoxaemia and hypercarbia. History of premature birth might have a bearing on this. Early detection of this from typical pigtail shaped signature capnogram enables the anaesthesiologists to treat the condition early in its course by application of PEEP.

The effectiveness of PEEP as the treatment modality corroborates the fact that neonates and small infants given weaning trial with endotracheal tube in situ also require CPAP to prevent airway closure due to endotracheal intubation.[6,9]

The clinical implication of this incidental finding is that it is highly recommended that capnography should be a routine monitor, not only for its usual role of confirmation of proper endotracheal tube placement, but also to detect airway closure at the earliest in small infants, especially sick ones.
